# Tertiary lymphoid tissue in early‐stage IgG4-related tubulointerstitial nephritis incidentally detected with a tumor lesion of the ureteropelvic junction: a case report

**DOI:** 10.1186/s12882-021-02240-1

**Published:** 2021-01-19

**Authors:** Tatsuhito Miyanaga, Keishi Mizuguchi, Satoshi Hara, Takeshi Zoshima, Dai Inoue, Ryo Nishioka, Ichiro Mizushima, Kiyoaki Ito, Hiroshi Fuji, Kazunori Yamada, Yuki Sato, Motoko Yanagita, Mitsuhiro Kawano

**Affiliations:** 1 Division of Rheumatology, Department of Internal Medicine, 13-1 Takaramachi, Kanazawa, Ishikawa Japan; 2 Department of Diagnostic Pathology, Kanazawa University Hospital, 13-1 Takaramachi, Kanazawa, Ishikawa Japan; 3 Department of Radiology, Kanazawa University Graduate School of Medical Science, 13-1 Takaramachi, Kanazawa, Ishikawa Japan; 4 Department of Hematology and Immunology, Kanazawa Medical University, 1-1 Daigaku, Uchinada, Kahoku, Ishikawa Japan; 5 Department of Nephrology, Kyoto University Graduate School of Medicine, Yoshidakonoe-cho, Sakyo-ku, Kyoto, Japan; 6 Medical Innovation Center TMK Project, Graduate School of Medicine, Kyoto University, 53 Shogoin, Kawahara-cho, Sakyo-ku, Kyoto, Japan; 7 Institute for the Advanced Study of Human Biology (ASHBi), Kyoto University, Yoshida-honmachi, Sakyo-ku, Kyoto, Japan

**Keywords:** IgG4-related kidney disease, IgG4-related tubulointerstitial nephritis, Tertiary lymphoid tissue

## Abstract

**Background:**

IgG4-related kidney disease causes renal impairment of unknown pathogenesis that may progress to kidney failure. Although ectopic germinal centers contribute to the pathogenesis of the head and neck lesions of IgG4-related disease, the presence of tertiary lymphoid tissue (TLT) containing germinal centers in IgG4-RKD has rarely been reported.

**Case presentation:**

We report a 72-year-old Japanese man who had IgG4-related tubulointerstitial nephritis (TIN) with TLT formation incidentally detected in a resected kidney with mass lesion of IgG4-related ureteritis in the ureteropelvic junction. During follow-up for past surgical resection of a bladder tumor, renal dysfunction developed and a ureter mass was found in the right ureteropelvic junction, which was treated by nephroureterectomy after chemotherapy. Pathology revealed no malignancy but abundant IgG4-positive cell infiltration, obliterative phlebitis and storiform fibrosis, confirming the diagnosis of IgG4-related ureteritis. In the resected right kidney, lymphoplasmacytes infiltrated the interstitium with focal distribution in the renal subcapsule and around medium vessels without storiform fibrosis, suggesting the very early stage of IgG4-TIN. Lymphocyte aggregates were also detected at these sites and consisted of B, T, and follicular dendritic cells, indicating TLT formation. IgG4-positive cells infiltrated around TLTs.

**Conclusions:**

Our case suggests that TLT formation is related with the development of IgG4-TIN and our analysis of distribution of TLT have possibility to elucidate IgG4-TIN pathophysiology.

## Background

IgG4-related kidney disease (IgG4-RKD) is the kidney lesion of IgG4-related disease (IgG4-RD) and its typical manifestation is plasma cell (PC)-rich tubulointerstitial nephritis (TIN) [1]. IgG4-RKD is usually suspected through kidney dysfunction with typical clinical features of IgG4-RD or characteristic radiological abnormalities of IgG4-related TIN (IgG4-TIN) [2]. Although IgG4-RKD responds well to glucocorticoids possibly through its immunosuppressive effect or its direct effect to resident fibroblasts [3], irreversible renal atrophic lesions often persist and progress to permanent renal damage [4, 5].

Differing from other TIN, a distinctive characteristic of IgG4-TIN is its spatial distribution [6–8]. Multiple low-density lesions on enhanced computed tomography (CT) are characteristic, and in histology, the border between involved and non-involved areas is sharply defined consistent with the radiological picture [6]. Lymphoplasmacytic infiltration often extends beyond the renal capsule, reflecting inflammation of extra-renal adipose tissue with IgG4-positive PCs (IgG4^+^PCs) [6, 7] and the “rim-like lesion” seen on enhanced CT. Analyses of autopsy specimens revealed lymphoplasmacytic infiltration distributed along medium-sized vessels in addition to subcapsular regions [8]. The factors underlying this characteristic distribution of IgG4-TIN, however, are unknown.

Ectopic germinal center (GC) formation is a major characteristic finding especially in the lacrimal and submandibular glands in IgG4-RD [9–12] while the lesion is relatively rare in IgG4-TIN [12–14]. Ectopic GCs are a component of tertiary lymphoid tissue (TLT) which is an accumulation of T cells, B cells, follicular dendritic cells and stromal cells accompanied by high endothelial venules and lymphatic vessels and develops in response to inflammation in organs outside the secondary lymphoid tissues [15–18]. TLT forms in various chronic inflammatory conditions such as autoimmune diseases, persistent infection, cancer, IgA glomerulonephritis, chronic graft rejection, and aging kidney [18–21]. In IgG4-TIN, however, report on ectopic GC in kidney parenchyma have been few.

We report here a patient with IgG4-related ureteritis whose resected kidney showed a very early stage of IgG4-TIN with TLT formation. TLT was distributed within the IgG4-TIN lesions which were located around medium-sized vessels and subcapsular regions. Hints obtained from these findings may help to elucidate the developmental mechanisms of IgG4-TIN.

## Case presentation

**Fig. 1:**
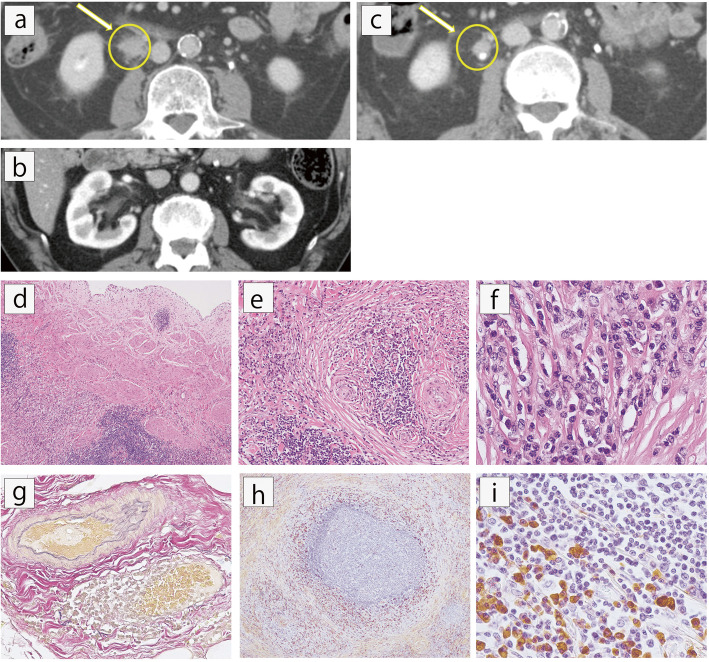
Radiology and histopathology of ureter mass in the ureteropelvic junction; **a** Contrast-enhanced CT showed ureter mass in the right ureteropelvic junction (arrow); **b** Right kidney presented mild hydronephrosis, while renal parenchyma showed no abnormalities; **c** Preoperative chemotherapy decreased the size of the ureter mass from 21 mm to 12 mm (arrow). The mass encased the ureter and had a smooth intraluminal surface of the ureter wall; **d** Histologically, mononuclear cells infiltrated outside the muscularis of the ureter [Hematoxylin-Eosin (HE) staining, × 40]; **e, f** Storiform fibrosis was observed [HE staining, (**e**) × 100, (**f**) × 400]; **g** Mononuclear cells also infiltrated the venules, indicating obliterative phlebitis (Elastica van Gieson staining, × 100); **h, i** Many IgG4-positive plasma cells infiltrated and surrounded germinal center-like structures [IgG4 immunostaining, (**h**) × 400, (**i**) × 40]

A 72-year-old Japanese man was admitted to our hospital because of progressive renal dysfunction after right nephroureterectomy for a right ureter mass. He had had type 2 diabetes since the age of 18 years and hypertension. Family history included diabetes in his father, mother and brothers. He had a smoking history of 150 pack-years. He did not have any allergies. His medications included amlodipine, sitagliptin phosphate hydrate, ipragliflozin L-proline, and insulin glargine. He had been followed for 18 years after surgical resection of a bladder tumor without recurrence. Six months previously, periodic laboratory examination had revealed mild renal dysfunction [serum creatinine 1.11 mg/dL, estimated glomerular filtration rate (eGFR) 50.7 ml/min/1.73 m^2^]. At this time, white blood cells 7,100/µL (eosinophil 241/µL), urinary protein (2+), occult blood (-), and urinary sugar (4+) were documented. Urine cytology was negative. Periodic abdominal CT revealed a mass lesion on his right upper ureter and mild right kidney hydronephrosis (Fig. 1a). On enhanced CT, it was 21 mm in diameter and surrounded the right upper ureter which had a smooth intraluminal surface.

Since no other radiological abnormalities were detected in either the renal parenchyma (Fig. 1b) or other organs, he was clinically diagnosed with right ureter cancer. As a neoadjuvant therapy, gemcitabine hydrochloride and cisplatin were administered, with on average 10 mg/day of intermittent dexamethasone also added as supportive therapy. Two months before admission, the mass became smaller (Fig. 1c) and then right nephroureterectomy was performed. Histopathology of the removed ureter mass revealed no malignancy, but IgG4^+^PC infiltration [> 100/high power field (HPF)], obliterative phlebitis and storiform fibrosis (Fig. 1d-h), which allowed a diagnosis of IgG4-related ureteritis. GC-like structures were also observed (Fig. 1i).

**Fig. 2:**
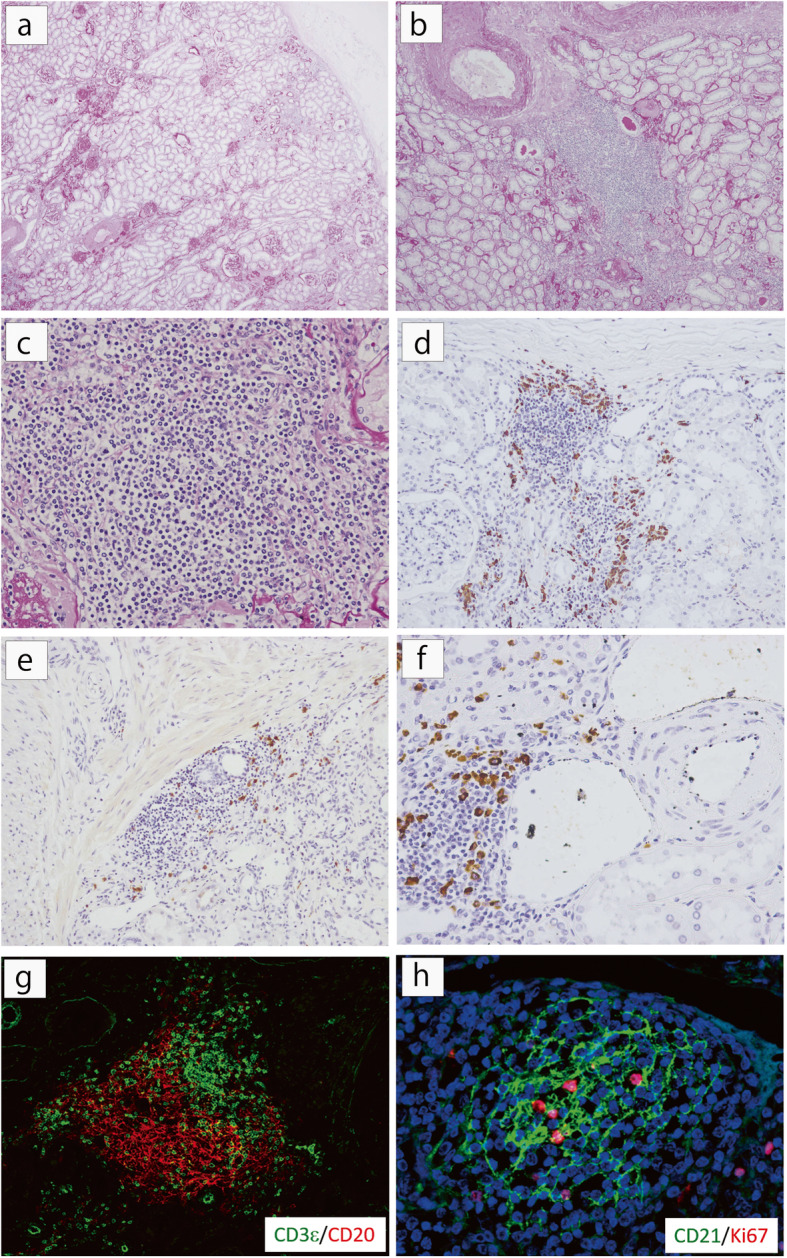
Histopathology of renal parenchyma in the concurrently removed right kidney. **a** Arteriosclerosis and arteriolar hyalinosis were found. About 30 % of glomeruli were globally sclerosed. Interstitial fibrosis was faint and no storiform fibrosis was noted [Periodic acid-Schiff (PAS) staining, × 20]. **b**, **c** Lymphoplasmacytic aggregates with scant fibrosis were observed focally in perivascular areas (arrow) [PAS staining, (**b**) × 40, (**c**) × 100]. **d** Lymphoplasmacytic infiltration was also detected in subcapsular areas. Germinal center (GC)-like structures surrounded by IgG4^+^ cells were located beneath the renal capsule (IgG4 immunostaining, × 100). **e**, **f** GC-like structures surrounded by IgG4-positive cells were also observed around interlobular arteries and venules [IgG4 immunostaining, (**e**) × 100, (**f**) × 200]. **g**, **h** Immunofluorescence revealed almost all GC-like structures to be aggregates of B and T cells and a CD21-positive follicular dendritic cell network, indicating mature tertiary lymphoid tissues (× 400)

Histopathology of the removed right kidney revealed arteriolar hyalinosis and arteriosclerosis of arcuate and interlobular arteries (Fig. 2a). Of 105 glomeruli, thirty-nine manifested global sclerosis. Remaining glomeruli had diffuse mesangial expansion, consistent with a diagnosis of diabetic glomerulosclerosis class IIa. In addition, many lymphoplasmacytes infiltrated only beneath the renal capsule and around medium-sized arteries and veins. Neither storiform fibrosis nor obliterative phlebitis was noted. In some parts, lymphoplasmacytes aggregated to form GC-like structures (Fig. 2b, c). Immunostaining showed abundant IgG4^+^PC infiltration (28/HPF) and IgG4^+^PCs surrounded the lymphatic follicle-like structures (Fig. 2d-f). Most of these structures were composed of T cells, B cells and CD21-positive follicular dendritic cells, suggesting the formation of mature TLT (Fig. 2g, h).

Since renal function gradually worsened after the operation, he was admitted to our department. Physical examination showed no swelling of lacrimal or salivary glands and no lymphadenopathy. Urinary protein was (±), urinary glucose (2+), and occult blood (-). Blood examination revealed white blood cells 6,900/µL (eosinophil 511/µL), kidney dysfunction (serum creatinine 2.04 mg/dL, eGFR 26.0 mL/min/1.73 m^2^), hemoglobin A1c 6.5 %, serum IgG 1,674 mg/dL, IgG4 112 mg/dL, anti-nuclear antibody 1:160, rheumatoid factor 47 IU/mL and normal complement levels (C3c 95 mg/dL, C4 22 mg/dL, CH50 52 U/mL), although these data would have been somewhat affected by the dexamethasone therapy before operation (Table 1). On enhanced CT, no other IgG4-RD lesions were detected. Considering that the right kidney had IgG4-TIN without imaging abnormality, we speculated that his left kidney also had IgG4-TIN and prescribed prednisolone (30 mg/day, 0.5 mg/kg/day) thereby preventing further deterioration of his renal function.

**Table 1:** Laboratory data of present case on admission to our hospital

Parameter	Value	Normal range
**Urinalysis**		
Protein	±	-
Occult blood	-	-
Sugar	2+	-
**Blood count**		
White blood cells (/μL)	6900	3,300-8,800
Eosinophil (/μL)	511	0-440
Red blood cells (×10^4^/μL)	412	430-550
Hemoglobin (g/dL)	11.7	13.5-17.0
Platelets (×10^4^/μL)	25.8	13.0-35.0
**Serum chemistry**		
Blood urea nitrogen (mg/dL)	21	8-22
Creatinine (mg/dL)	2.04	0.60-1.00
Uric acid (mg/dL)	6.8	3.6-7.0
Sodium (mEq/L)	140	135-149
Potassium (mEq/L)	4.5	3.5-4.9
Chlorine (mEq/L)	112	96-108
Alkaline phosphatase (IU/L)	398	115-359
Gamma-glutamyltransferase (IU/L)	16	10-47
Aspartate aminotransferase (IU/L)	10	13-33
Alanine aminotransferase (IU/L)	7	8-42
Lactate dehydrogenase (IU/L)	156	119-229
Amylase (IU/L)	103	4-113
Total protein (g/dL)	7.4	6.7-8.3
Albumin (g/dL)	4.0	4.0-5.0
Hemoglobin A1c (NGSP) (%)	6.5	4.3-5.8
**Immunological findings**		
C-reactive protein (mg/dL)	0.1	0.0-0.3
IgG (mg/dL)	1674	870-1,700
IgG4 (mg/dL)	112	<135
IgA (mg/dL)	232	110-410
IgM (mg/dL)	142	33-190
IgE (IU/mL)	419	<250
CH50 (U/mL)	52	32-47
C3 (mg/dL)	95	65-135
C4 (mg/dL)	22	13-35
Anti-nuclear antibody	x160	-
Pattern	nucleolar	
Rheumatoid factor (IU/mL)	47	<20

*Abbreviation*: *Ig* Immunoglobulin

## Discussion and conclusions

The present case showed a ureter mass in the right ureteropelvic junction suggestive of a ureteral malignancy for which ureteronephrectomy was performed. Histopathological analysis showed IgG4-ureteritis and the coexistence of the very early stage of IgG4-TIN with TLT formation in the renal parenchyma.

In this case, IgG4-TIN was incidentally detected without any suggestive radiological findings on contrast-enhanced CT. Various radiological abnormalities such as multiple low-density lesions and rim-like lesion on contrast-enhanced CT have been found in IgG4-RKD, each of which reflects the respective pathological findings [2, 6]. On the other hand, few cases of IgG4-TIN have been reported to not manifest any radiological abnormalities on contrast-enhanced CT [4, 22]. In one of them, severe hypocomplementemia was the sole clue to possible renal involvement [22]. Therefore, it is extremely difficult to analyze the very early stage of IgG4-TIN histopathologically because imaging abnormalities generally appear in the moderately advanced stage of the disease and a renal biopsy is usually not performed in the absence of imaging abnormalities or decreased renal function. In this context, the present case is of particular interest because the very small and restricted area of lymphoplasmacytic infiltration and scant fibrosis indicated that it had the very early stage of IgG4-TIN. Although glucocorticoid administered before nephrectomy might have reduced the TLT size and numbers [23], scant fibrosis without imaging features of IgG4-TIN supports the contention that this case had very early disease. This might provide hints to the pathophysiological mechanisms underlying the kidney lesion of IgG4-RD and to the uneven spread of inflammation in the kidney.

Notably, in the analysis of the scattered kidney lesions in this case, we found that the distribution of the lesions of IgG4-TIN was consistent with the area in which TLT often forms in the kidney, i.e. around perivascular and subcapsular areas. TLT functions as local sites of antigen presentation, clonal expansion, lymphocyte activation and class switching in B cells [15–17]. Although TLT formation may be either deleterious or protective according to the context [18], TLTs in kidney diseases are usually detrimental [18–20]. TLT formation predicts a poor renal outcome in IgA nephropathy [19] and leads to graft loss due to chronic rejection [20]. After acute kidney injury, the presence of TLTs contributes to the progression to end-stage kidney disease in the elderly [18]. In IgG4-RD, ectopic GC contributes to the pathogenesis of IgG4-RD mediated by the induction of ectopic GC formation by type 2 follicular T cells, which in turn promotes the differentiation of naïve B cells into plasmablasts and PCs, and IgG4 class-switching *in situ* in IgG4-RD [24]. Although ectopic GC frequently form in head and neck lesions [9, 25], very few cases with IgG4-TIN accompanied by GC formation in kidney lesions have been reported [12–14]. Similarly, the frequency of ectopic GCs is rare and their number is small in the pancreas and retroperitoneal lesions [26]. TLT formation reflects the immune response against locally displayed antigens [21]. In addition, we previously found identical IgG4-CDR3 sequences in all of salivary gland, lung, and peripheral blood, suggesting the existence of common antigen(s) shared by patients with IgG4-RD [27]. Considering the fact that ectopic GCs are frequently formed at salivary and lacrimal glands which are the most frequently involved organs in IgG4-RD [28], we speculate that the initial antigen delivery and antigen presentation by antigen presenting cells occur at these sites while stimulated B cells or plasmablasts move from lacrimal or salivary glands to other organs such as the kidney and contribute to the formation of the multiple organ lesions of this disease. For this reason, the present case is valuable in documenting that TLT can be detected in the renal cortex at a very early stage of IgG4-TIN. Notably, TLT was located beneath the renal capsule and around medium-sized vessels, which are characteristic and frequent sites of development of IgG4-TIN [8]. Given that the lymphatic flow is rich near the renal capsule and around medium-sized vessels [29], the development of the extra-renal-capsular and perivascular lesions of IgG4-TIN could be explained by the findings of the present case. Taken together, our findings suggest that IgG4-TIN develops and distributes in tandem with the formation of TLT beneath the renal capsule and around medium-sized vessels, culminating in the development of interstitial inflammation associated with IgG4^+^PC infiltration. Although TLT could be a potential marker of relapse and resistance to treatment in IgG4-RD [30], its clinical role remains undetermined due to scant data available in support of this theory. Further study is clearly needed to clarify the clinical role of TLT in IgG4-TIN.

In conclusion, we report a case of the very early stage of IgG4-TIN containing TLT which was incidentally detected concurrently with IgG4-related ureteritis in the ureteropelvic junction. The distribution of TLT in this case was consistent with that of IgG4-TIN, and IgG4-positive cells infiltrated around TLT, suggesting that IgG4-TIN develops and distributes in tandem with TLT formation. Clarification of the role of TLT in IgG4-TIN lesion formation would facilitate understanding of IgG4-TIN pathophysiology.

## Data Availability

All the data relevant to this report are included in the manuscript.

## Abbreviations

TLT: Tertiary lymphoid tissue
IgG4-RKD: IgG4-related kidney disease
IgG4-TIN: IgG4-related tubulointerstitial nephritis
CT: Computed tomography
GC: Germinal center
eGFR: Estimated glomerular filtration rate
PC: Plasma cell
IgG4^+^PCs: IgG4-positive plasma cells
HPF: High-power field

## References

[1] Yamaguchi T, Kanetsuna Y, Honda K (2012). Characteristic tubulointerstitial nephritis in IgG4-related disease. Hum Pathol.

[2] Kawano M, Saeki T, Nakashima H (2011). Proposal for diagnostic criteria for IgG4- related kidney disease. Clin Exp Nephrol.

[3] Iguchi T, Takaori K, Mii A (2018). Glucocorticoid receptor expression in resident and hematopoietic cells in IgG4-related disease. Mod Pathol.

[4] Saeki T, Kawano M, Mizushima I (2013). The clinical course of patients with IgG4-related disease. Kidney Int.

[5] Mizushima I, Yamamoto M, Inoue D (2016). Factors related to renal cortical atrophy development after glucocorticoid therapy in IgG4-related kidney disease: a retrospective multicenter study. Arthritis Res Ther.

[6] Kawano M, Saeki T, Nakashima H (2019). IgG4-related kidney disease and retroperitoneal fibrosis: An update. Mod Rheumatol.

[7] Inoue K, Okubo T, Kato T (2018). IgG4-related stomach muscle lesion with a renal pseudotumor and multiple renal rim-like lesions: A rare manifestation of IgG4-related disease. Mod Rheumatol.

[8] Hara S, Kawano M, Mizushima I (2016). Distribution and components of interstitial inflammation and fibrosis in IgG4-related kidney disease: analysis of autopsy specimens. Hum Pathol.

[9] Zen Y, Nakamura Y (2010). IgG4-Related Disease A Cross-sectional Study of 114 Cases. Am J Surg Pathol.

[10] Maehara T, Moriyama M, Nakashima H (2012). Interleukin-21 contributes to germinal centre formation and immunoglobulin G4 production in IgG4-related dacryoadenitis and sialoadenitis, so-called Mikulicz’s disease. Ann Rheum Dis.

[11] Satoh-Nakamura T, Kurose N, Kawanami T (2015). CD14 + follicular dendritic cells in lymphoid follicles may play a role in the pathogenesis of IgG4-related disease. Biomed Res.

[12] Ebbo M, Daniel L, Pavic M (2012). IgG4-related systemic disease. Features and treatment response in a French cohort: results of a multicenter registry. Medicine.

[13] Pozdzik AA, Brochériou I, Demetter P (2012). Azathioprine as successful maintenance therapy in IgG4-related tubulointerstitial nephritis. Clin Kidney J.

[14] Yoshita K, Kawano M, Mizushima I (2012). Light-microscopic characteristics of IgG4-related tubulointerstitial nephritis: distinction from non-IgG4-related tubulointerstitial nephritis. Nephrol Dial Transplant.

[15] Neyt K, Perros F, GeurtsvanKessel CH, Hammad H, Lambrecht BN (2012). Tertiary lymphoid organs in infection and autoimmunity. Trends Immunol.

[16] Van de Pavert SA, Meibius RE (2010). New insights into the development of lymphoid tissues. Nat Rev Immunol.

[17] Ruddle NH (2014). Lymphatic vessels and tertiary lymphoid organs. J Clin Invest.

[18] Sato Y, Mii A, Hamazaki Y (2016). Heterogeneous fibroblasts underlie age-dependent tertiary lymphoid tissues in the kidney. JCI Insight.

[19] Pei G, Zeng R, Han M (2014). Renal interstitial infiltration and tertiary lymphoid organ neogenesis in IgA nephropathy Clin. J Am Soc Nephrol.

[20] Thaunat O, Patey N, Caligiuri G (2010). Chronic rejection triggers the development of an aggressive intragraft immune response through recapitulation of lymphoid organogenesis. J Immunol.

[21] Pipi E, Nayar S, Gardner DH (2018). Tertiary lymphoid structures: Autoimmunity goes local. Front Immunol.

[22] Otani M, Morinaga M, Nakajima Y (2015). IgG4-related kidney disease in which the urinalysis, kidney function and imaging findings were normal. Intern Med.

[23] Sato Y, Boor P, Fukuma S, et al. Developmental stages of tertiary lymphoid tissue reflect local injury and inflammation in murine and human kidneys. Kidney Int. 2020 in press. 10.1016/j.kint.2020.02.023 32473779

[24] Akiyama M, Suzuki K, Yasuoka H (2018). Follicular helper T cells in the pathogenesis of IgG4-related disease. Rheumatology.

[25] Yamamoto M, Takahashi H, Shinomura Y, Umehara H, Okazaki K, Stone JH, Kawa S, Kawano M (2012). Lacrimal gland and salivary gland lesions. IgG4-related disease.

[26] Ichiro M, Kasashima S, Fujinaga Y (2019). Clinical and Pathological Characteristics of IgG4-Related Periaortitis/Periarteritis and Retroperitoneal Fibrosis Diagnosed Based on Experts’ Diagnosis. Ann Vasc Dis.

[27] Kakuchi Y, Yamada K, Ito K (2016). Analysis of IgG4-positive clones in affected organs of IgG4-related disease. Mod Rheumatol.

[28] Wallace ZS, Zhang Y, Perugino CA (2019). Clinical phenotypes of IgG4-related disease: an analysis of two international cross-sectional cohorts. Ann Rheum Dis.

[29] Russell PS, Hong J, Windsor JA (2019). Renal lymphatics: anatomy, physiology, and clinical implications. Front Physiol.

[30] Touzani F, Pozdzik A (2019). New insights into immune cells cross-talk during IgG4-related disease. Clin Immunol.

